# Machine learning-based risk prediction model for cognitive dysfunction in elderly individuals

**DOI:** 10.1371/journal.pone.0336058

**Published:** 2025-12-19

**Authors:** Lei Zhang, Xuan Xiang, Wei Chen, Haijun Miao, Ting Zou, Ruikai Wu, Xiaohui Zhou

**Affiliations:** Department of Geriatrics, First Affiliated Hospital of Xinjiang Medical University, Urumqi, Xinjiang, China; Federal University Dutse, NIGERIA

## Abstract

**Background:**

With the advancement of globalization, the prevalence of cognitive dysfunction in the elderly population has risen significantly. Early intervention may dramatically alleviate the disease burden and reduce economic costs associated with cognitive impairment. This study aims to construct a risk prediction model for cognitive dysfunction based on machine learning (ML) algorithms, providing healthcare professionals and patients with a more accurate and effective tool for risk assessment.

**Methods:**

This study included 1,325 elderly participants who completed cognitive assessments and comprehensive laboratory blood tests. Risk factors for cognitive dysfunction were identified through univariate analysis, multivariate logistic regression, LASSO regression, and the Boruta algorithm. Nine ML methods—Random Forest (RF), Light Gradient Boosting Machine (LightGBM), Extreme Gradient Boosting (XGBoost), Logistic Regression, K-Nearest Neighbor (KNN), Support Vector Machine (SVM), Artificial Neural Network (ANN), Decision Tree, and Elastic Net—were employed to construct the prediction models. The Shapley Additive Explanations (SHAP) algorithm was utilized to interpret the final model.

**Results:**

The Random Forest model exhibited the highest predictive performance, with an AUC value exceeding those of other models. SHAP analysis identified age, race, education level, diabetes, and depression as the primary predictors of cognitive dysfunction in the elderly. The calibration curve indicated a strong alignment between the model’s predictions and actual outcomes, while the decision curve confirmed the model’s clinical applicability.

**Conclusion:**

Age, race, education level, diabetes, and depression are significant influencing factors of cognitive dysfunction in the elderly. Among the ML algorithms evaluated, the Random Forest model exhibited the best predictive performance.

## 1. Introduction

The global population is aging rapidly, and increased life expectancy has made cognitive dysfunction in the elderly a major public health concern [1]. Cognitive impairment not only diminishes cognitive function and quality of life but also imposes substantial disease and economic burdens on patients and their families [2–4]. According to the World Alzheimer Report, an estimated 46.8 million individuals worldwide were affected by dementia in 2015, with projections indicating a rise to 131.5 million by 2050. [5]. Every three seconds, someone is diagnosed with dementia, and the annual cost of dementia is estimated at $1 trillion, a figure expected to double by 2030. Alzheimer’s disease (AD) is the most common form of dementia [6]. Primary prevention of AD holds significant potential, as one-third of global AD cases are attributable to modifiable risk factors. The World Health Organization (WHO) Guidelines for Risk Reduction of Cognitive Decline and Dementia [7] and the 2020 Lancet Commission report [8] identified several modifiable risk factors for dementia, including low education, advanced age, smoking, excessive alcohol consumption, obesity, depression, physical inactivity, hearing impairment, hypertension, diabetes, social isolation, traumatic brain injury, and air pollution.

Growing evidence suggests a link between cognitive dysfunction and inflammation [9]. Immunosenescence and inflammaging are hallmark features of aging [10], with inflammation playing a pivotal role in the pathogenesis of cognitive decline and dementia [11–14]. The systemic immune-inflammation index (SII) and systemic inflammation response index (SIRI), recently developed composite inflammatory markers, are widely used to assess systemic inflammation [15,16]. Studies by Wang et al.[17–22] have demonstrated a significant association between SII, SIRI, and cognitive impairment. Insulin resistance (IR) is also significantly associated with an increased risk of cognitive decline [23]. The triglyceride-glucose (TyG) index, derived from fasting triglyceride (TG) and blood glucose (FBG) levels, is a cost-effective and readily available surrogate marker for IR [24]. Research [25–28] indicates that a higher TyG index is significantly correlated with an elevated risk of dementia. Investigating the relationship between SII, SIRI, the TyG index, and cognitive function may provide a basis for early detection of cognitive impairment.

With the rapid advancement of artificial intelligence, risk models incorporating demographic, behavioral, and psychosocial factors have emerged. Machine learning (ML) offers unique advantages in medical prediction by automatically identifying key predictors and their interactions through feature importance ranking and decision-splitting mechanisms [29]. However, existing studies are often limited to single-algorithm validation, lacking multi-model performance comparisons and interpretability analyses. Therefore, this study leverages data from the 2011–2014 National Health and Nutrition Examination Survey (NHANES) to explore risk factors for cognitive dysfunction in the elderly. By employing nine ML algorithms to construct predictive models and comparing their performance using calibration and decision curve analyses, this study aims to identify the optimal model, offering new insights for healthcare professionals in predicting cognitive impairment risk among the elderly.

## 2. Materials and methods

### 2.1 Study population

**Fig 1:**
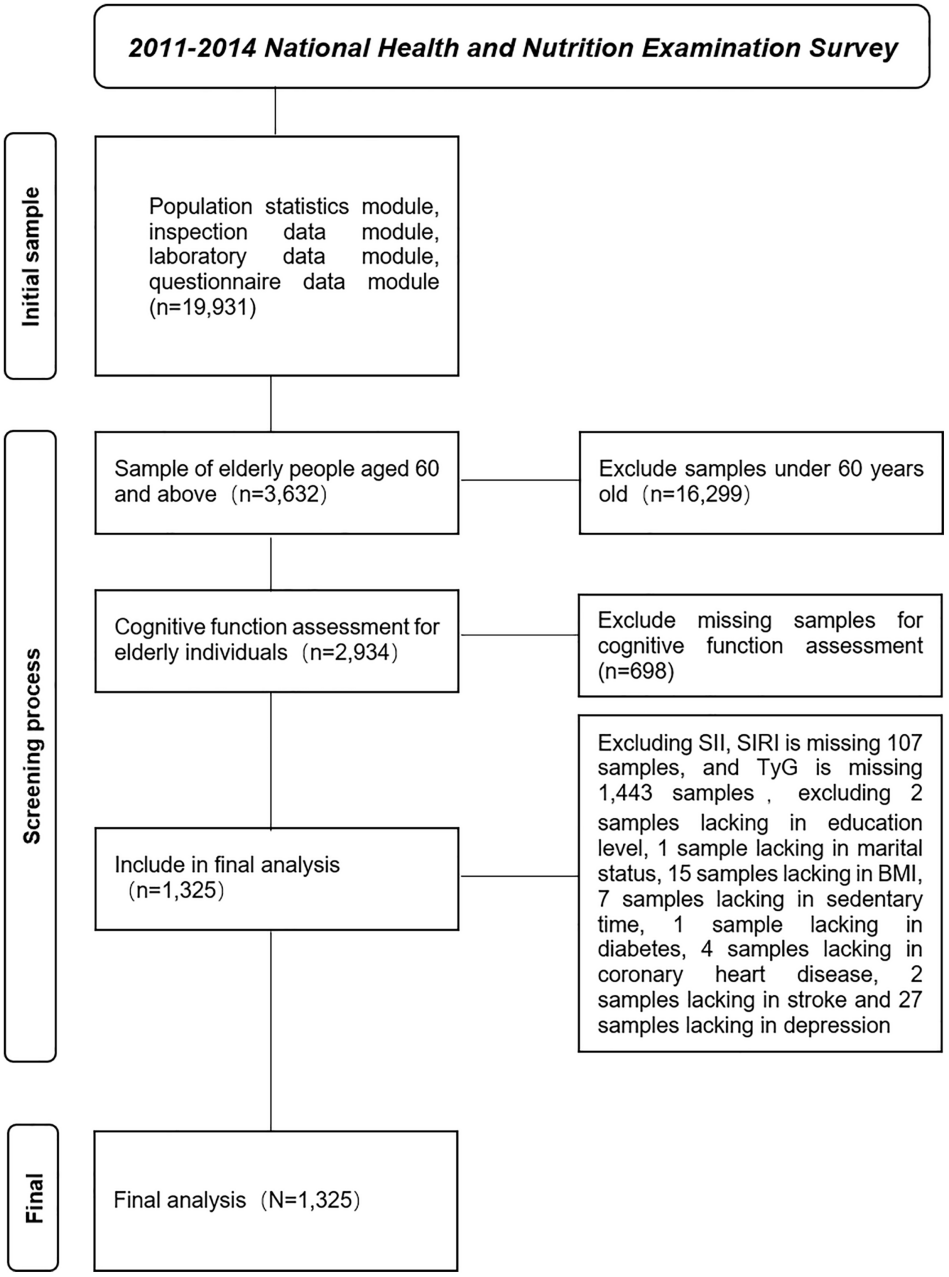
Flowchart of participant selection.

This study utilized cross-sectional data from the 2011–2014 National Health and Nutrition Examination Survey (NHANES), conducted by the National Center for Health Statistics (NCHS) and the Centers for Disease Control and Prevention (CDC) to assess the health and nutritional status of individuals across all age groups in the United States. The survey was approved by the NCHS Ethics Review Board, and all participants provided written informed consent (https://www.cdc.gov/nchs/nhanes/about/erb.html?CDC_AAref_Val=https://www.cdc.gov/nchs/nhanes/irba98.htm). Inclusion criteria were: (1) age ≥ 60 years; and (2) complete responses to all survey items. A total of 1,325 elderly participants were included in the final analysis (Fig 1).

### 2.2 Study variables and definitions

#### 2.2.1 Cognitive function assessment.

Cognitive ability in participants aged ≥60 years was evaluated using three standardized tests:

1. Consortium to Establish a Registry for Alzheimer’s Disease (CERAD): Including immediate recall (CERAD-IR) and delayed recall (CERAD-DR) tests.
2. Animal Fluency Test (AFT).
3. Digit Symbol Substitution Test (DSST).

These tools are widely used in studies analyzing cognitive function and its risk factors [30–32]. A composite Z-score, termed the Overall cognitive ability score, was calculated by averaging the standardized scores of the CERAD, AFT, and DSST tests [33–36]. Although no definitive threshold for cognitive impairment has been established in prior studies, the 25th percentile of the Overall cognitive ability score was used as the cutoff in this study [37–39]. Participants were categorized into two groups: normal cognitive function and cognitive impairment.

#### 2.2.2 Depression.

The Patient Health Questionnaire-9 (PHQ-9), a self-report tool widely used in clinical practice and research, was employed to screen, diagnose, and assess depression. The questionnaire consists of nine items covering core depressive symptoms, including low mood, loss of interest, sleep disturbances, appetite changes, fatigue, feelings of worthlessness, poor concentration, psychomotor retardation, and suicidal ideation. Each item is scored from 0 (“not at all”) to 3 (“nearly every day”), with a maximum total score of 27. A PHQ-9 score ≥10 was considered indicative of depression [40].

#### 2.2.3 Immune-inflammatory indices and triglyceride-glucose index.

The systemic immune-inflammation index (SII), systemic inflammation response index (SIRI), and triglyceride-glucose (TyG) index were calculated using complete blood count (CBC) laboratory results from the NHANES database [18]. The following measurements were used (reported in 1,000 cells/μL or mg/dL): platelet count (PC), neutrophil count (NC), monocyte count (MC), lymphocyte count (LC), triglycerides (TG), and fasting blood glucose (FPG). The indices were derived as follows:

1. SII = (platelet count × neutrophil count)/lymphocyte count;
2. SIRI = (neutrophil count × monocyte count)/lymphocyte count.
3. TyG=In[fasting triglycerides (TG, mg/dL) X fasting blood glucose (FPG, mg/dL)/2]

#### 2.2.4 Covariates.

Based on study design requirements, variables were assessed across different dimensions, with categorical variables assigned numerical values. Binary variables were coded as 0 or 1, while multi-category variables were assigned incremental values (e.g., 0, 1, 2). The selected covariates included:

Continuous variables: Age, minutes of sedentary activity.

Categorical variables: Gender (male, female); Race/ethnicity (Mexican American, Other Hispanic, Non-Hispanic White, Non-Hispanic Black, Other Race—including multiracial); Education level (<9th grade, 9–11th grade [including 12th grade without diploma], high school graduate/GED or equivalent, some college or AA degree, college graduate or above); Marital status (married, widowed, divorced, separated, never married, living with partner); BMI (<25, 25– < 30, ≥ 30); Self-reported sleep trouble (no, yes); Diabetes (no, borderline, yes); Heart disease (no, yes); Stroke (no, yes); Depression (no, yes).

### 2.3 Model development

Risk factors for cognitive impairment were screened using univariate analysis, multivariate logistic regression, Least Absolute Shrinkage and Selection Operator (LASSO) regression, and the Boruta algorithm. The dataset was randomly split into training (70%) and testing (30%) sets. Nine supervised machine learning (ML) algorithms were employed to construct prediction models:

1. Random Forest (RF); 2. Light Gradient Boosting Machine (LightGBM); 3. Extreme Gradient Boosting (XGBoost); 4. Logistic Regression; 5. K-Nearest Neighbor (KNN); 6. Support Vector Machine (SVM); 7. Artificial Neural Network (ANN); 8. Decision Tree; 9. Elastic Net; Hyperparameter optimization is crucial for model performance. We employed grid search with 5-fold cross-validation to identify the optimal hyperparameter combinations for each algorithm. The hyperparameter space for each algorithm is detailed in Table 1. The configuration yielding the best average performance across the folds was selected for model building. (Table 1).

**Table 1:** Hyperparameter values for each machine learning algorithm.

Algorithms	Hyperparameters	Explanations	Search Space	Optimal Value
**KNN**	n_neighbors	Number of K neighbors	{1, 2, 3, 4, 5, 6, 7,8,9,10}	1
	p	Power parameter of distance measurement (Minkowski distance)	{1, 2}	1
	weights	Weight functions used in prediction	{uniform, distance}	uniform
**SVM**	C	regularization parameter	{0.001, 0.01, 0.1, 1, 10, 100}	100
	gamma	Kernel function parameters	{0.001, 0.01, 0.1, 1, 10, 100}	1
	kernel	kernel function type	{linear, rbf, sigmoid}	rbf
**ANN**	hidden_layer_sizes	Number of hidden layer neurons	{1, 2,..., 32}	27
	max_iter	Maximum Number Of Iterations	{500, 1000, 1500}	500
**Decision Tree**	max_depth	The maximum depth of a tree	{2, 3, 4, 5, 6, 7, 8, None}	None
	min_samples_split	The minimum number of samples required to split internal nodes	{2, 3,..., 10}	4
	min_samples_leaf	Minimum number of samples required on leaf nodes	{1, 2, 3, 4, 5}	1
	criterion	A function for measuring the quality of splitting	{gini, entropy}	entropy
	max_features	The number of features to consider when searching for the best segmentation	{auto, sqrt, log2, None}	None
**LightGBM**	learning_rate	learning rate	{0.01, 0.05, 0.1}	0.05
	num_leaves	The maximum number of leaves per tree	{31, 40, 50}	50
	max_depth	The maximum depth of a tree	{−1, 5, 10}	10
	n_estimators	Iteration times (number of trees)	{100, 200, 300}	200
	subsample	Subsample ratio	{0.8, 0.9, 1.0}	0.9
	colsample_bytree	Column sampling ratio for each tree	{0.8, 0.9, 1.0}	1
	reg_alpha	L1 regularization	{0, 0.1, 0.5}	0.1
	reg_lambda	L2 regularization	{0, 0.1, 0.5}	0
**Random Forest**	n_estimators	The number of trees in the forest	{10, 20,..., 200}	40
	max_depth	The maximum depth of a tree	{None, 3, 4,..., 13}	10
	min_samples_split	The minimum number of samples required to split internal nodes	{2, 3,..., 10}	3
	min_samples_leaf	Minimum number of samples required on leaf nodes	{1, 2, 3, 4, 5}	2
	max_features	The number of features to consider when searching for the best segmentation	{auto, sqrt, log2, None, 1,...}	log2
**XGBoost**	learning_rate	learning rate	{0.01, 0.05, 0.1}	0.01
	max_depth	The maximum depth of a tree	{3, 6, 9}	9
	n_estimators	The number of trees	{50, 100, 200}	100
	subsample	Subsample ratio	{0.8, 0.9, 1.0}	0.9
	colsample_bytree	Column sampling ratio for each tree	{0.8, 0.9, 1.0}	0.8
**Elastic Net**	C	regularization strength	{0.001, 0.01, 0.1, 1, 10, 100}	0.01
	l1_ratio	Regularization ratio of L1 and L2	{0.1, 0.3, 0.5, 0.7, 0.9}	0.3
**Logistic Regression**	C	The reciprocal of the regularization strength	{1e-4, 1e-3,..., 1e + 4}	0.01
	penalty	Specify the penalties used in regularization	{l1, l2, None}	l2
	solver	Algorithms used for optimizing problems	{liblinear, lbfgs}	lbfgs

### 2.4 Evaluation metrics

Performance metrics included accuracy, recall, specificity, positive predictive value (PPV), negative predictive value (NPV), area under the receiver operating characteristic curve (AUC-ROC), and F1-score. Calibration curves were used to assess model consistency, with the Brier score quantifying calibration performance (lower scores indicate better accuracy: 0–0.1 = excellent, 0.1–0.25 = good, > 0.25 = poor). Decision curve analysis (DCA) evaluated clinical utility, and the Shapley Additive Explanations (SHAP) algorithm interpreted feature contributions to model predictions.

### 2.5 Statistical analysis

Analyses were conducted using *R 4.3* and *Python 3.11.5*. Non-normally distributed continuous variables were expressed as median (interquartile range) [*M*(*P*_25_, *P*_75_)], with group comparisons performed using nonparametric tests. Categorical variables were reported as frequencies and percentages (n, %), with group comparisons assessed via Z-tests. A two-tailed *P* < 0.05 was considered statistically significant.

## 3. Results

### 3.1 Participant characteristics

Among the 1,325 participants, 1,092 (82.42%) had normal cognitive function, while 233 (17.58%) exhibited cognitive impairment. Significant differences (*P* < 0.05) were observed between groups for age, race, education level, marital status, diabetes, stroke, and depression. No significant differences (*P* > 0.05) were found for sedentary activity, SII, SIRI, TyG index, gender, BMI, self-reported sleep trouble, or heart disease (Table 2).

**Table 2:** Basic characteristics of study participants (n = 1325).

Variables	Total (n = 1325)	Non-cognitive impairment group (n = 1008)	Cognitive impairment group (n = 317)	Statistic	*P*
Age, *M*(*P*_25_, *P*_75_)	69.00(64.00,76.00)	67.00(63.00,74.00)	73.00(66.00,80.00)	Z = −7.592	**<0.001**
Minutes sedentary activity, *M*(*P*_25_, *P*_75_)	360.00(240.00,480.00)	360.00(240.00,480.00)	360.00(240.00,480.00)	Z = −1.346	0.178
SII, *M*(*P*_25_, *P*_75_)	440.75(318.50,642.21)	442.57(320.57,629.50)	434.45(306.31,702.80)	Z = −0.637	0.524
SIRI,*M*(*P*_25_, *P*_75_)	1.08(0.72,1.60)	1.06(0.71,1.53)	1.14(0.72,1.73)	Z = −1.433	0.152
TyG,*M*(*P*_25_, *P*_75_)	8.64(8.27,9.07)	8.63(8.24,9.06)	8.67(8.34,9.11)	Z = −1.723	0.085
Gender, n(%)				χ² = 3.020	0.082
Male	650(49.06)	481(47.72)	169(53.31)		
Female	675(50.94)	527(52.28)	148(46.69)		
Race/Hispanic origin, n(%)				χ² = 76.251	**<0.001**
Mexican American	117(8.83)	72(7.14)	45(14.20)		
Other Hispanic	135(10.19)	77(7.64)	58(18.30)		
Non-Hispanic White	672(50.72)	563(55.85)	109(34.38)		
Non-Hispanic Black	270(20.38)	185(18.35)	85(26.81)		
Other Race – Including Multi-Racial	131(9.89)	111(11.01)	20(6.31)		
Education level, n(%)				χ² = 301.940	**<0.001**
Less than 9th grade	149(11.25)	42(4.17)	107(33.75)		
9-11th grade (Includes 12th grade with no diploma)	187(14.11)	111(11.01)	76(23.97)		
High school graduate/GED or equivalent	317(23.92)	243(24.11)	74(23.34)		
Some college or AA degree	375(28.30)	333(33.04)	42(13.25)		
College graduate or above	297(22.42)	279(27.68)	18(5.68)		
Marital status, n(%)				χ² = 32.456	**<0.001**
Married	784(59.17)	615(61.01)	169(53.31)		
Widowed	243(18.34)	161(15.97)	82(25.87)		
Divorced	157(11.85)	124(12.30)	33(10.41)		
Separated	29(2.19)	14(1.39)	15(4.73)		
Never married	70(5.28)	60(5.95)	10(3.15)		
Living with a partner	42(3.17)	34(3.37)	8(2.52)		
BMI, n(%)				χ² = 1.076	0.584
< 25	366(27.62)	274(27.18)	92(29.02)		
25 ≤ - < 30	462(34.87)	359(35.62)	103(32.49)		
≥ 30	497(37.51)	375(37.20)	122(38.49)		
Ever told the doctor had trouble sleeping, n(%)				χ² = 0.285	0.593
No	933(70.42)	706(70.04)	227(71.61)		
Yes	392(29.58)	302(29.96)	90(28.39)		
Diabetes, n(%)				χ² = 35.861	**<0.001**
No	969(73.13)	769(76.29)	200(63.09)		
Borderline	59(4.45)	51(5.06)	8(2.52)		
Yes	297(22.42)	188(18.65)	109(34.38)		
Heart disease, n(%)				χ² = 1.754	0.185
No	1192(89.96)	913(90.58)	279(88.01)		
Yes	133(10.04)	95(9.42)	38(11.99)		
Stroke, n(%)				χ² = 6.040	**0.014**
No	1232(92.98)	947(93.95)	285(89.91)		
Yes	93(7.02)	61(6.05)	32(10.09)		
Depression, n(%)				χ² = 28.234	**<0.001**
No	1197(90.34)	935(92.76)	262(82.65)		
Yes	128(9.66)	73(7.24)	55(17.35)		

### 3.2 Feature selection

**Fig 2:**
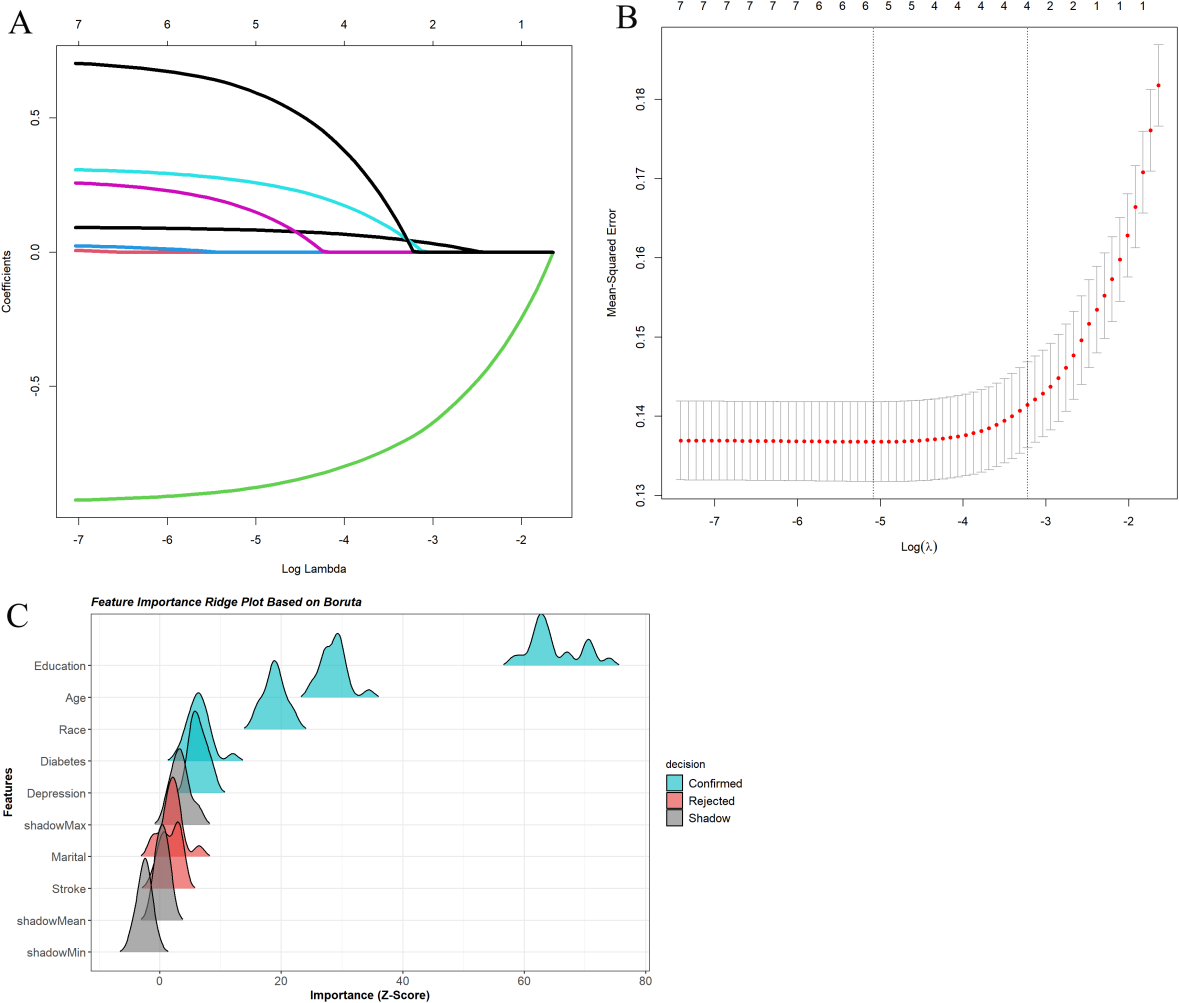
Lasso regression and Boruta algorithm for screening predictive factors. **(A)** Coefficient path of Lasso regression. **(B)** Cross-validation results of Lasso regression. **(C)** Boruta algorithm results.

Use multiple logistic regression, Lasso regression, and the Boruta algorithm to screen risk factors closely related to cognitive impairment in the elderly, and include variables with statistically significant differences in univariate analysis. The five important predictive factors obtained from multiple logistic regression, Lasso regression, and Boruta algorithm are Age, Race, Education level, Diabetes, and Depression (Table 3, Fig 2).

**Table 3:** Multivariate logistic analysis.

*Variables*	*β*	*S.E*	*Z*	*P*	*OR (95%CI)*
Intercept	−8.099	1.004	−8.069		
Age	0.126	0.014	8.781	**<0.001**	1.134(1.102 ~ 1.166)
Race/Hispanic origin					
Mexican American					1.000(Reference)
Other Hispanic	0.737	0.336	2.194	**0.028**	2.090(1.082 ~ 4.038)
Non-Hispanic White	−0.725	0.308	−2.356	**0.018**	0.485(0.265 ~ 0.885)
Non-Hispanic Black	0.646	0.315	2.047	**0.041**	1.907(1.028 ~ 3.539)
Other Race – Including Multi-Racial	−0.300	0.391	−0.767	0.443	0.741(0.344 ~ 1.594)
Education level					
Less than 9th grade					1.000(Reference)
9-11th grade (Includes 12th grade with no diploma)	−1.358	0.274	−4.959	**<0.001**	0.257(0.150 ~ 0.440)
High school graduate/GED or equivalent	−2.143	0.270	−7.941	**<0.001**	0.117(0.069 ~ 0.199)
Some college or AA degree	−2.976	0.285	−10.427	**<0.001**	0.051(0.029 ~ 0.089)
College graduate or above	−3.486	0.345	−10.091	**<0.001**	0.031(0.016 ~ 0.060)
Marital status					
Married					1.000(Reference)
Widowed	0.099	0.203	0.489	0.625	1.104(0.742 ~ 1.643)
Divorced	0.070	0.268	0.262	0.794	1.073(0.634 ~ 1.815)
Separated	0.567	0.466	1.217	0.224	1.764(0.707 ~ 4.401)
Never married	−0.655	0.431	−1.520	0.128	0.519(0.223 ~ 1.209)
Living with a partner	0.149	0.511	0.292	0.771	1.161(0.426 ~ 3.159)
Diabetes					
No					1.000(Reference)
Borderline	−0.530	0.473	−1.120	0.263	0.589(0.233 ~ 1.488)
Yes	0.596	0.178	3.337	**<0.001**	1.814(1.279 ~ 2.574)
Stroke					
No					1.000(Reference)
Yes	0.300	0.282	1.063	0.288	1.349(0.776 ~ 2.345)
Depression					
No					1.000(Reference)
Yes	0.696	0.250	2.791	**0.005**	2.006(1.230 ~ 3.272)

### 3.3 Performance comparison of nine prediction models

**Fig 3:**
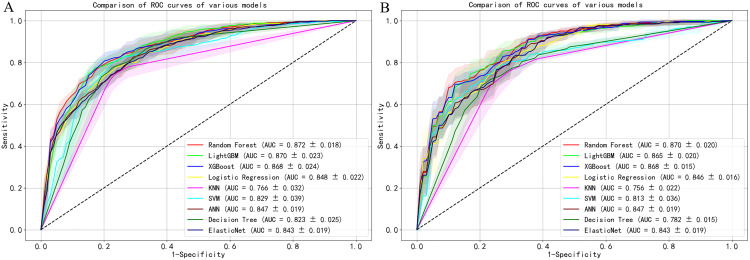
ROC curves of 9 machine learning prediction models. **(A)** Training set. **(B)** Test set.

The nine ML algorithms were trained using the selected predictors. Random Forest demonstrated the highest performance: Training set: AUC = 0.872 (95% CI: 0.854–0.890), accuracy = 0.787, sensitivity = 0.795, specificity = 0.780, PPV = 0.786, NPV = 0.792, F1-score = 0.789. Testing set: AUC = 0.870 (95% CI: 0.850–0.890), accuracy = 0.770, sensitivity = 0.778, specificity = 0.762, PPV = 0.768, NPV = 0.775, F1-score = 0.772 (Table 4, Fig 3).

**Table 4:** Performance comparison of 9 machine learning prediction models.

Model	ROC AUC	Accuracy	Sensitivity	Specificity	Positive Predictive Value	Negative Predictive Value	F1 Score
Random Forest	0.872	0.787	0.795	0.780	0.786	0.792	0.789
	0.870	0.770	0.778	0.762	0.768	0.775	0.772
LightGBM	0.870	0.784	0.795	0.773	0.781	0.791	0.787
	0.865	0.770	0.765	0.776	0.775	0.770	0.769
XGBoost	0.868	0.790	0.783	0.797	0.797	0.788	0.788
	0.868	0.762	0.778	0.746	0.756	0.772	0.766
Logistic Regression	0.848	0.765	0.751	0.779	0.774	0.758	0.761
	0.846	0.764	0.761	0.766	0.764	0.764	0.763
KNN	0.766	0.766	0.766	0.766	0.766	0.768	0.765
	0.756	0.755	0.785	0.726	0.743	0.772	0.762
SVM	0.829	0.788	0.809	0.767	0.777	0.800	0.793
	0.813	0.765	0.778	0.752	0.760	0.774	0.768
ANN	0.847	0.752	0.735	0.769	0.762	0.745	0.747
	0.847	0.755	0.742	0.769	0.763	0.752	0.751
Decision Tree	0.823	0.770	0.765	0.776	0.774	0.767	0.769
	0.782	0.739	0.722	0.756	0.746	0.732	0.734
ElasticNet	0.843	0.762	0.758	0.766	0.765	0.762	0.760
	0.843	0.759	0.755	0.763	0.762	0.758	0.758

**Fig 4:**
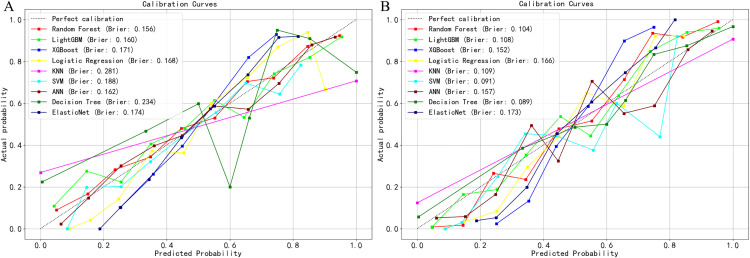
Calibration curves of 9 machine learning prediction models. **(A)** Training set. **(B)** Test set.

On the test set, Random Forest LightGBM XGBoost Logistic Regression KNN SVM ANN Decision Tree Elastic Net, evaluate the accuracy and clinical practicality of the model. The calibration curve of the test set shows that the Brier scores of all 9 models are below 0.20, indicating that the 9 models have good accuracy and the predicted results are consistent with the actual results (Fig 4).

**Fig 5:**
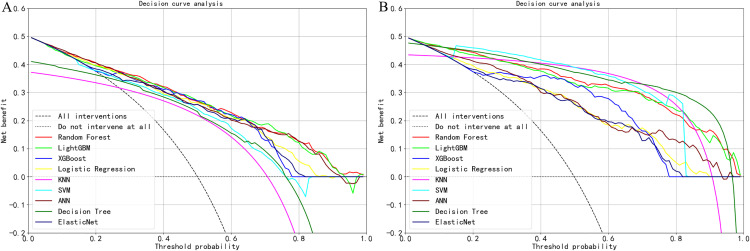
DCA curves of 9 machine learning prediction models. **(A)** Training set. **(B)** Test set.

The DCA curve showed that when the risk threshold was between 0.1 and 0.8, Random Forest LightGBM XGBoost Logistic Regression KNN SVM ANN Decision Tree,Elastic Net the model can obtain better clinical net benefit, indicating that the model has better clinical applicability (Fig 5).

Based on the above analysis, the Random Forest model performs the best in predicting the risk of cognitive impairment in the elderly, with high prediction accuracy and good clinical practicality. Therefore, the Random Forest model was chosen as the final model for predicting the risk of cognitive impairment in the elderly.

### 3.4 Feature importance analysis

**Fig 6:**
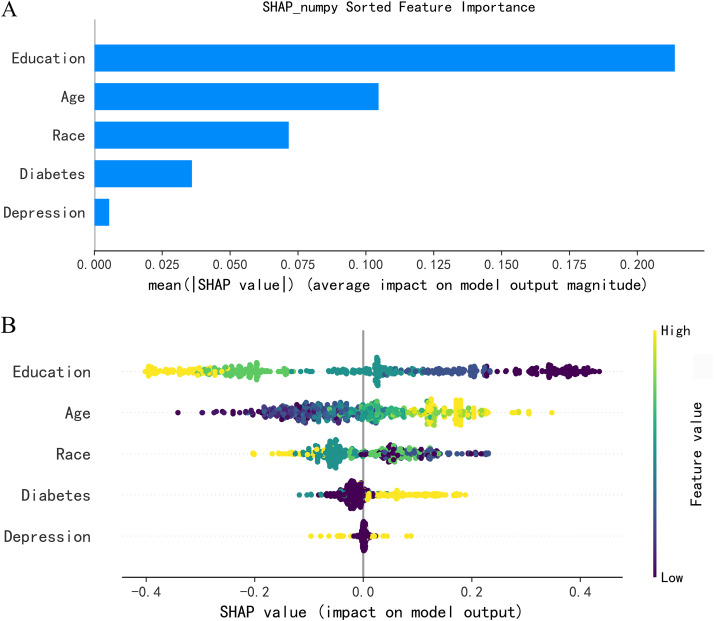
SHAP’s Visual Explanation of the Global Model. **(A)** Bar chart. **(B)** Bee colony chart.

SHAP analysis of the Random Forest model ranked predictor importance as: education level > age > race > diabetes > depression (Fig 6A). Swarm plots revealed negative associations between education level and cognitive impairment, and positive associations for age, diabetes, and depression (Fig 6B).

**Fig 7:**
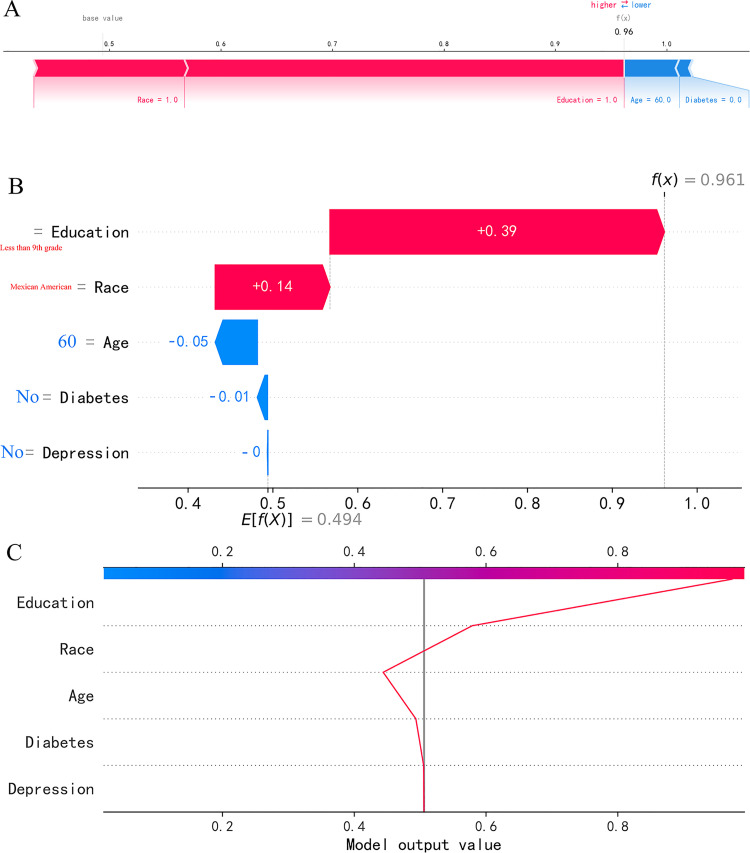
Visual interpretation of SHAP for single-sample cases. **(A)** Force Plot. **(B)** Waterfall Plot. **(C)** Decision Plot.

Single-sample SHAP diagrams, waterfall diagrams, and decision diagrams can explain the prediction results of a single case. For example, the data from Case 1 shows that the Education level is Less than 9th grade, Race is Mexican American, Age is 60 years old, no diabetes, no depression, and the Random Forest risk model predicts a probability of 0.96 for the risk of cognitive impairment in the elderly (Fig 7).

## 4 Discuss

In recent years, machine learning (ML), deep learning, artificial intelligence, and statistical analysis have been increasingly applied to medical research [41–45]. ML algorithms can leverage large datasets for training, thereby enhancing the accuracy and predictive power of models. These algorithms autonomously learn patterns and relationships from data to construct predictive models without manual intervention, improving efficiency. Moreover, they can continuously update and optimize models with new data, ensuring adaptability to evolving environments and datasets [46]. ML excels at processing high-dimensional data and complex relationships, uncovering nonlinear associations and patterns that may elude traditional methods. Its capacity to handle large-scale data enables the extraction of actionable insights, while its interpretability clarifies model mechanics and decision-making processes, offering precise predictive and decision-support tools in medicine [47].

During model development, nine ML algorithms were evaluated, with the Random Forest (RF) model demonstrating superior performance in predicting cognitive impairment risk among elderly individuals. As a classical ML algorithm, RF efficiently handles high-dimensional data by employing an ensemble of decision trees, which mitigates overfitting and captures complex feature interactions [48–50]. Its robustness to noise and outliers further enhances reliability in real-world applications. To improve transparency, the Shapley Additive Planations (SHAP) algorithm was employed for model interpretation. Globally, SHAP quantified the relative contribution of each feature to cognitive impairment risk; locally, it elucidated how individual predictors influenced specific cases. This dual interpretability strengthens the model’s clinical utility [51].

Feature or variable selection is the core of predictive model development [52,53]. This study used three algorithms, namely multi-factor logistic regression, Lasso regression, and Boruta algorithm, to obtain Age, Race, Education level, Diabetes, and Depression predictive factors. Based on the five predictive factors, a risk prediction model was constructed, which has good predictive performance, accuracy, and clinical benefits. Previous studies have focused on age [54,55] Education level [56,57] Race [58] Diabetes [59],Depression [44]. The literature indicates that the predictive factors we have chosen are available and reliable.

Through the SHAP algorithm, it was found that education level is the primary predictor, and a higher education level can reduce the risk of cognitive impairment. Education can help improve memory, cognitive stimulation, and cognitive abilities [60]. Cognitive stimulation activities may slow down the rate of hippocampal atrophy during normal aging [61], and may even prevent the accumulation of amyloid plaques [62]. The deposition of amyloid beta (Aβ) is a biomarker for cognitive impairment. Education mainly strengthens the control of processes and the understanding of concepts in cognitive function. Compared with those with shorter education periods, those with longer education periods have an 85% lower risk of Mild Cognitive Impairment (MCI) and Alzheimer’s disease [63]. The cognitive reserve hypothesis [64] proposes that stimulating the environment promotes the growth of new neurons in the form of neurogenesis, thereby promoting neural plasticity. With the improvement of cultural level and the increase of cognitive reserve, the expression of cognitive decline may be delayed [65].

Cognitive guidelines and expert consensus point out [66–69] that age is one of the predictive factors for the risk of cognitive impairment. As the body gradually ages, various organs and tissues begin to age, and the functional connections of the brain network will selectively weaken, inevitably leading to a decline in cognitive ability. Lee et al. [70,71] found that hippocampal neurons located deep in the temporal lobe of the brain help us classify and understand human perception and experience from the most basic to highly complex things. As we age, the balance between pattern separation and pattern completion is disrupted, and memory is impaired. Moreover, the hippocampus is highly susceptible to hypoxia/ischemic damage, and the function of the hippocampal vascular system is crucial for maintaining neurocognitive health. The decrease in hippocampal blood flow occurs during healthy aging and can lead to neuronal atrophy and memory decline in the hippocampus [72,73].

There is a close relationship between diabetes and cognitive impairment [74]. Type 2 diabetes can increase the incidence of Alzheimer’s disease (AD) by 1.5–2.5 times [71]. Many scholars have found that diabetes and cognitive impairment share many common pathophysiological bases. Diabetes can cause an inflammatory reaction, metabolic disorder, microvascular disease, oxidative stress, A β deposition, neurofibrillar tangle, leading to insulin resistance, damage to synaptic plasticity, synaptic degeneration, and cell death. There are abnormal insulin signal transduction pathways, weakened mitochondrial function, autonomic nervous dysfunction, and neurocellular inflammation in diabetes patients, which can affect the brain tissue and structure, and ultimately lead to cognitive decline [75,76].

The reasons why depression increases the risk of cognitive impairment involve multiple mechanisms at the neurobiological, endocrine, and behavioral levels. Firstly, depression leads to abnormal levels of key neurotransmitters such as serotonin and dopamine in the brain, impairing synaptic transmission efficiency and directly affecting memory encoding and cognitive flexibility [77]. Long-term depression may interfere with glutamate-mediated neuronal excitability, inhibit hippocampal neural plasticity, and accelerate cognitive decline [78]. Depressive states activate microglia, promote the release of pro-inflammatory factors such as IL-6 and TNF-α, damage neurons, and hinder synaptic remodeling [79]. Depression leads to hyperfunction of the hypothalamic pituitary adrenal (HPA) axis, sustained elevation of cortisol, and direct toxicity to hippocampal neurons [80]. Depression is often accompanied by abnormal glucose metabolism, and an increase in the TyG index reflects insulin resistance, which can reduce brain glucose utilization and impair cognitive function [29]. Depression related stress hormones can promote abnormal phosphorylation of tau protein, accelerate the formation of neurofibrillary tangles, and are directly associated with cognitive symptoms of Alzheimer’s disease [81]. There is a bidirectional relationship between cognitive impairment and depression [82]. On the one hand, cognitive impairment leads to a decrease in social participation and emotional regulation ability, which in turn triggers depression and exacerbates depressive symptoms [83,84]. On the other hand, depression accelerates cognitive decline by promoting neuroinflammation and abnormal brain function, and this vicious cycle accelerates the transition to dementia.

Previous studies have found that as the SII, SIRI, and TyG indices increase, the risk of cognitive impairment in the elderly increases [14,18,85–92]. However, after cleaning the null values of complete blood count (CBC) in NHANES laboratory tests from 2011 to 2014, this study did not find any direct statistical differences in SII, SIRI, and TyG between the cognitively normal and cognitively impaired elderly groups during analysis. Considering the cleaning of missing values may result in a small sample size, which may reduce the power of the test and prevent the detection of actual differences. Secondly, SII and SIRI inflammatory markers mainly reflect systemic inflammatory status, but their specificity in cognitive impairment may not be high. Although inflammation is associated with cognitive decline, SII and SIRI indices are not sensitive biomarkers for cognitive impairment, especially in the elderly population, which is influenced by multiple confounding factors such as coexisting chronic diseases and drug treatment. The TyG index evaluates insulin resistance. Although there is a theoretical association between insulin resistance and cognitive impairment (such as Alzheimer’s disease), in the actual population, individual variations in metabolic factors (such as lifestyle and genetic background) may dilute the differences between the cognitively normal group and the cognitively impaired group. This also leads to the lack of statistical significance of SII, SIRI, and TyG as predictive factors in this study.

There are certain limitations to this study. Firstly, the sample size of SII, SIRI, and TyG indices is relatively small after cleaning the laboratory to check the complete blood count (CBC) null value, which limits the learning ability of ML; Secondly, there are shortcomings in feature selection, which fail to fully explore all potential factors that affect the risk of cognitive impairment in the elderly; The third issue is that model selection and parameter tuning need to be optimized. The above factors have led to significant room for improvement in indicators such as AUC and recall rate of our research model, although they are within an acceptable range. In future research, it is expected to expand the sample size of indices such as SII, SIRI, TyG, etc. to improve testing efficiency, and combine multimodal evaluation (such as imaging and genetic markers) to reduce confounding bias. At the same time, more efficient algorithms will be explored to improve model performance and expand the applicability of the model.

In summary, Age, Race, Education level, Diabetes, and Depression are the influencing factors of cognitive impairment in the elderly. This study constructs a prediction model for cognitive impairment risk in the elderly based on machine learning algorithms. Among them, the random forest algorithm has the best prediction performance and certain predictive value, which can provide new ideas and methods for early identification and intervention of cognitive impairment risk in the elderly.
